# Time pressure in surgical teams, a help or a hindrance to patient safety?

**DOI:** 10.1016/j.heliyon.2025.e41967

**Published:** 2025-01-15

**Authors:** Annet van Harten, Theo J.H. Niessen, Jur J. Koksma, Hein G. Gooszen, Tineke A. Abma

**Affiliations:** a Dept. of Process Improvement and Innovation, Radboud University Medical Center, F.C. Donderslaan 2, 6525, GJ Nijmegen, the Netherlands; b Faculty of Nursing, Fontys University of Applied Sciences, Ds. Th. Fliednerstraat 2, 5631, BN Eindhoven, the Netherlands; c Dept. of Research in Learning and Education, Radboud University Medical Center, Gerard van Swietenlaan 2, 6525, GB Nijmegen, the Netherlands; d Dept. of Lung Diseases, Radboud University Medical Centre, Geert Grooteplein Zuid 10, 6525, GA Nijmegen, the Netherlands; e Dept. Ethics, Law and Humanities, AmsterdamUMC, De Boelelaan 1089a, 1081, HV Amsterdam, the Netherlands

**Keywords:** Time pressure, Habit theory, Mindful routines, Patient safety, Operating room teamwork, Situational awareness

## Abstract

**Background:**

Patient safety requires mindful routines in the operating room. Usually, time pressure is presented as an unavoidable constraint to mindful routines and a consequence of workload imposed on teams. We aim to understand time pressure and how it interacts with developing mindful routines.

**Methods:**

This naturalistic case study was conducted with a surgical team in a Dutch academic hospital using ethnographic methods including participant observation, interviews, and fieldnotes. The researcher observed the team for 103 h. Our analysis integrates habit theory and mindful organising principles.

**Results:**

Team culture reflected deference to speed, preoccupation with productivity, conflict avoidance, and value on affective relationships. Conflicting priorities arose from differences in safety norms, worries about time, and beliefs about what saves time. Addressing these conflicting priorities, however, was rare. Creating shared Situational Awareness (SA) helped prevent or mitigate time pressure, though it was not a consistently embedded routine. New routines were often compromised under time pressure, while established habits showed resilience to time constraints.

**Conclusions:**

Rather than being workload-driven, time pressure emerged as a co-constructed outcome of conflicting priorities and the preservation of affective relationships. The imperative to save time motivated shared situational awareness and the formation of new mindful routines. We recommend enhancing mindful routines by refining current practices in mortality and morbidity meetings, expanding stakeholder involvement, and addressing prevailing concerns.

## Highlights

• Time pressure within the operating team is co-created by avoiding conflict over priorities.
• Saving time motivated developing situational awareness and a new mindful routine.
• We recommend fostering mindful routines by enhancing existing routines.
• Mortality and morbidity meetings can be optimised by addressing preoccupations with time.
• Making mortality and morbidity meetings interprofessional could further enhance effectiveness.

## 1 Introduction

Over the past two decades, healthcare organisations worldwide have introduced numerous initiatives, programmes, and tools aimed at reducing preventable patient harm. While patient safety was defined as the absence of harm, it is now seen as an active capability rooted in both system robustness and human behaviours [[1], [2], [3]]. Adaptability and resilience have become recognised as essential capacities, enabling effective responses to unexpected events within an increasingly complex environment shaped by demographic changes, technological advancements, and specialised practices. Evidence suggests that hospitals with lower mortality rates do not necessarily experience fewer errors but are more adept at recovery and rescue [4,5]. The patterns of thought and action that underpin this adaptability are known as mindful practice [6]. However, hospitals fail to turn periodic mindful practice into a consistent enduring habit of action and thought [6]. Surgical complication rates, for example, have remained largely unchanged over the past two decades [5]. In other words, hospitals fail to make the transition from episodic mindful practice to enduring mindful routines. Such routines might include performing safety checks with fidelity, habitual cross-monitoring during procedures, and inviting to speak up.

Research suggests that a key barrier to this transition lies in the increasing focus on cost efficiency and revenue by senior managers, which contributes to time pressure [5,7]. At the team level, frontline workers often attribute suboptimal checklist performance and quality improvements to time pressure—the sense of having too many demands on limited time [8]. The sense of time pressure can be caused by a high workload, but not necessarily [9].

Most quality and safety improvement studies regard time pressure as an unavoidable constraint within which individuals must perform reliably to ensure patient safety [1,2]. Consequently, many studies focus on developing individual resilience and competence to handle substantial workloads [[10], [11], [12]] and on mitigating adverse effects like fatigue and burnout [13,14]. In contrast, we propose a novel perspective: we view time pressure not as an inevitable barrier to patient safety, nor solely as a matter of individual competence, but rather as a dynamic factor in developing mindful routines.

The purpose of this article is to understand time pressure and how it interacts with the development of mindful routines in the context of patient safety.

## 2 Methods

### 2.1 Research team reflexivity

The research team comprised five authors from diverse backgrounds: change management and psychology, medical humanities, educational sciences, surgery, and nursing studies. All data were collected by Mrs A. van Harten, the lead researcher, who acted as a senior change facilitator and crew resource management (CRM) trainer. As such she was familiar with the field and several participants of the specialty of the case. She had a trusting relationship with the management of the operating rooms and anaesthesia and gained trust from the management of the surgeons by presenting her plans and intentions on two occasions. During the CRM training she introduced herself and her motivations. The core team was informed in more detail about the theoretical and methodological background of the researcher.

Given the importance of strong relationships with participants before, during, and after the study, there was a risk of biased observations and interpretations. However, as a trained consultant, coach, and psychologist, Mrs van Harten was equipped to reflect on her emotions, power dynamics, and interests. Throughout the study, she recorded reflections in a diary and discussed her role and observations in biweekly sessions with Dr L. Fluit who deliberately acted as a critical friend asking questions about presumptions and methodological decisions [15].

### 2.2 Setting

The study was done in a Dutch academic hospital. The case entails the interprofessional surgical team of one specialty (approximately 45 persons) involving all surgeons, residents, operating room (OR) nurses, anaesthetists and anaesthesia nurses. The surgeons were mostly male the nurses mostly female. The anaesthesia nurses and physicians were mixed male and female.

The team enrolled in a CRM training programme involving 1 h of e-learning followed by a one-day group training. The training aimed to enhance awareness of their own human fallibility, risky team behaviours, and safe practices. Some surgeons viewed the CRM training as an externally imposed obligation. A core team, formed from team representatives, was tasked with improving work methods. They prioritised enhancing Situational Awareness (SA) through daily briefings at the start of the day and encouraging "speak-up". These briefings—distinct from the pre-incision time-out—enabled the team to know each other, align on daily planning, and discuss patient procedures, risks, and resource availability. While familiar to the anaesthetists and nurses, this routine was new to most surgeons.

Each surgical specialty has a multidisciplinary workplace management team (comprising a nurse, anaesthetist, and surgeon) that coordinates weekly operating room schedules. Members of this team also participated in the core team.

### 2.3 Study design

A naturalistic case study design [16] suits the explorative aim of the study. This means that we studied the setting in-depth to understand a single demarcated entity. This multidisciplinary surgical team, chosen for their commitment to implementing Crew Resources Management (CRM) and a new mindful routine (a daily briefing), provided an ideal context for studying time pressure and its relation to mindful routines. Furthermore, we wanted to study time pressure in a surgical specialty with almost only elective patients instead of a specialty with many acute patients. This surgical team met that requirement. This way the case offered most learning potential, one of the main selection criteria in case study research [16,17]. This academic hospital was chosen, because the researcher was familiar with the culture and procedures of the operating rooms as a result of her position as a consultant in this hospital.

Data were collected by the first author over ten months through participant observations in the operating room, handovers, morbidity and mortality meetings, and CRM training sessions; semi-structured interviews with key stakeholders; and informal conversations (see Table 1). Observations and self-reflections were recorded in a field diary. The stakeholders interviewed at the start of the study were selected to gain insight in: all perspectives on safety, their perceptions of their influence, their interest in and comments on the (preliminary) research question. These open interviews lasted approximately 1 h each.

**Table 1:** Data collection.

Activity	Hours, numbers	Role and Method	Data recording
Stake holder interviews with key players of the following departments: Surgical specialty, Operation Rooms, Anaesthesia, Recovery, Quality and safety, Crew Resource Management (CRM) program, National Healthcare Inspection	8 h, 10 interviews	Semi structured interviews with topic list.	Transcribed audio recordings
CRM trainings with OR nurses, anaesthesia nurses, surgeons, anaesthetists, management surgeons	24 h, 3 groups	(co)-trainer, Participant observations	Fieldnotes
OR observations	42 h	Observations	Fieldnotes
Informal conversations	8 h	Unstructured interviews	Fieldnotes and sometimes transcribed audio recording
Core team meetings (first meeting 1 day)	15 h, 8 meetings	Facilitator, Participant observations	Transcribed audio recordings, minutes and fieldnotes
Attendance of patient hand over and complication meetings	6 h, 4 meetings	Observations (fly on the wall)	Fieldnotes
e-mails, telephone calls	Not counted		Fieldnotes

We studied time pressure as a subjective experience of individuals and groups rather than time spent objectively; therefore, we did not measure time quantitatively. Furthermore, we studied the development of observable mindful routines and, as mentioned in the introduction, took as a premise that the routines contribute to patient safety.

Analysis was conducted in phases through an iterative process. Field notes and transcripts were read by all co-authors and Dr L. Fluit and discussed in three research team meetings, the first held halfway through data collection. Using Jackson and Mazzei's "thinking with theory" method [18], we initially diverged perspectives by "plugging in" theories to explore unexpected details and uncover new interpretations. which provided valuable insights for addressing the research question. In the third meeting, the researchers converged to two supporting fields of knowledge: mindful organising [2,19] and habit theory [6,20,21] which provided valuable insights for addressing the research question.

To enhance the study's trustworthiness, we employed prolonged engagement, researcher reflexivity, member checking, and thick descriptions to ensure credibility and transferability. Appendix S1 provides further methodological details.

## 3 Results

The results are structured around five key team dynamics (see Table 2). Three of these dynamics illuminate the creation of time pressure: deference to speed, preoccupation with productivity, and the avoidance of conflict while pursuing conflicting priorities. The remaining two dynamics demonstrate how team members either prevented or responded to time pressure: by creating SA and by skipping new routines and adhering to established ones.

**Table 2:** Quotes and vignettes per theme.

Deference to speed	
Q1	*‘I always feel rushed, especially at the start of the day. We can start only in one room at the same time, and yet the second room is always annoyed when we show up 8.05am.’ - Anaesthetist Susan*
V1	Vignette 1. Being fast as a source of respect At the end of the working day researcher XXX is seated on the couch in the restaurant of the OR complex with a cup of coffee. Anaesthetist Bernhard, familiar to her, comes in and takes place next to her for a chat. At some point, they bring up the farewell of a mutually known colleague anaesthetist. *XXX: ‘Did he decline a big farewell feast because he felt he had received too little recognition from his colleagues?’* *Anaesthetist Bernhard: ‘Indeed. He meant a lot for the department and the hospital, especially in the field of quality and safety. A lot of people comment on that because he is not a fast hero on the floor in the OR and he is wordy. But so what? He realised a lot of valuable initiatives that we would not have accomplished without him. […] How important is it that you are fast?’* *XXX: ‘Is being handy and fast necessary to gain respect from your colleagues?’*
Preoccupation with productivity	
V2	Vignette 2. Starting on time Several weeks after starting the implementation of the briefing, the main concern of the core team is how much time the briefing consumes. *Jennifer (OR-nurse): ‘Things are improving, but it takes quite some time before everybody is present for the briefing. If we perform the briefing, and then the time-out, then we're seeing the first activity in the theatre at 8.20 a.m.! [again, with emphasis] 8.20 a.m.! That's really too late in my opinion.’* *Jeroen (surgeon): ‘We've got the charts with late starts and early endings. You can see that start-up time has slowly been moving back to normal since the introduction of the briefing.’* *Jorin (resident): ‘I often have the impression that the anaesthesia consultant is eager to attend the briefing. While with us, a surgeon consultant often or sometimes doesn't attend the briefing. George doesn't show up before the knife is in the patient and then the resident is allowed to start.’* Jeroen: ‘I'd start at 8.00 *a.m.* with the team available at that time, with as many people present as possible. So, then you have to have sort of minimal requirements. Staff members must be there as much as possible, or you're going to be wasting time needlessly. We'll be unable to motivate a few of our staff members. For some, it's been the habit for many years not to show up in the theatre before the resident has made the incision.’
Q2 Q3	*‘We strongly feel that for a good surgeon clinical work comes first and research is the second assignment. All other tasks are of lower priority. No other department in this hospital does as much clinical work as we do.’- Medical head of the surgeons Bert* *‘We have a large supply of patients, so it is more that we receive a lot than that we push to produce a lot. Really, we produce too much, so rather not. We have a certain expertise, a large front door and the conviction and ambition that we are the best for those patients. So, we are not going to refer them to someone else, and then the solution is to keep one's shoulder to the wheel.’- Manager Saskia*
Avoiding conflict and pursuing conflicting priorities	
Q4 Q5	*“They walk the extra mile for you if they like you”. – Surgeon Kees* *“Performing the briefing contributes to the feeling of being a team.’ – Surgeon Sophie*
V3	Vignette 3. Nurses safeguarding ending in time. *Jennifer (senior OR nurse): ‘The other day, it was really one of those days, you know. At the start of the day, it was already Murphy's law. So, at eleven o'clock my colleague said: “We really won't be going to make it before 4 p.m.” So, I said: “You're right, we won't make it if we're going to do everything he (the surgeon) says. But we're not going to bring this up any sooner than when we've finished this, because otherwise we'll only get grumbling and discord.” She said: “All right, are you going to say it?” “Yes, I will.” So, at a suitable moment, when the patient had to be repositioned on the table, I said: “One thing, now or later, but I want us to look realistically at the programme for today and decide what's going to be done and what not. Then we can all agree on that, and we won't mention it again the rest of the day. If we must work overtime, we'll settle now who will be the one because I get really annoyed if people ask every 2 h “How much longer will it take?”* *Then the surgeon said:” I'll try to reschedule the programme with the other rooms.” So, he went off. When he came back, he said: “It's been arranged.” I said: “All right, when this patient is off the table, I want to hear how we're going to do it and how we'll divide the tasks the rest of the day.” So, we did at the sign-out, when the patient was still asleep, and everybody agreed. So, in my room there wasn't any grumbling anymore because I knew what to do and so did my colleagues.’* *Researcher, XXX: “This would be a really good example to share with your colleagues! Do you ever do so?”* *Jennifer: “No, that's of no use; it's in your character and in your age. When I would be 20 years younger, I wouldn't have done it either. Now I have the position and the guts to do this.”*
Creating team SA to handle time pressure	
V4	Vignette 4. The quick surgeon George enters the OR and takes a moment to overview the room. Then he says in a cheerful way to the anaesthesia nurse *‘Hi Toon, fellow, how are you?’* He asks the anaesthetist *‘Do you want to advance today? Then, we will take care of that. The next operation will be done by Anton. so that will probably take 6 h I'm afraid.’* In this boastful but cordial tone, he has small conversations with most team members. The OR nurse whispers with a smile to the observer, *‘With him we will surely be ready on time, he is really fast’*. Resident Arie gives a short recapitulation of the briefing and the sign in and shortly thereafter incision starts. During the operation George is looking around regularly and he stays in contact with the anaesthesia team about blood loss etcetera. At every stage during the operation (removal of organs for example) he asks whether all materials and all team members are ready for the next stage in a clear voice, and he only progresses when he hears their confirmations. By doing so every team member has awareness of the situation. In a small conversation with observer XXX the anaesthesia nurse says: ‘*Even when there is a bleeding, you can ask him questions. He goes on communicating very well, so you always know where he is heading for, and I can make myself clear where we are heading for.’*
Skipping new routines and adhering to old routines	
Q6 Q7	*‘There should be a certain format for such a meeting. For example, it is strange that the resident should always present a medical complication. Why? Then it becomes such an obligation, and there are already so many obligations. The next thing is that it is completely free what you want to discuss. If it would be a discussion about what could we have done differently, then that could be interesting. Now we present an article we found about the same treatment and where happened this and that. That is nice but not very instructive. So, I would think: less often, interdisciplinary [with nurses and anaesthesiologists] and a good format.’- Resident Bram* *‘I tell myself that the time out is really useful because it is not done to omit it. But if I'm honest with myself, I do not really believe it contributes to patient safety.’- Surgeon Johan*

### 3.1 The creation of time pressure

#### 3.1.1 Deference to speed

A predominant aspect of the team culture was a strong deference to speed. The prevailing belief was that “a good surgeon is a quick surgeon.” Delays in induction adversely impacted the average operating time, defined as the interval from the start of induction to the closure of the wound. Consequently, some surgeons exhibited irritability and restlessness when induction took longer than anticipated, even during periods of low workload. The waiting period induced a sense of pressure for both those waiting and those being waited on (quote 1). This deference to speed was evident across all professions, including anaesthetists (vignette 1).

#### 3.1.2 Preoccupation with productivity

Another significant cultural aspect was a preoccupation with productivity, defined as the amount of work completed within a given timeframe. This preoccupation was reflected in the managerial language used by Nurse Jennifer and Surgeon Jeroen when discussing operating room occupancy (vignette 2). It was also reflected in Jeroen's tendency to weigh the opinions of fellow surgeons, some of whom considered the briefing a waste of time, against the nurses' perspective, who viewed the briefing as a time-saving measure. The briefing helped the nurses in anticipating on required materials later in the day. Most surgeons were benevolent towards the new routine due to its potential to expedite processes and because their surgical specialty was among the last to adopt this routine.

Quote 2 from the medical head illustrates that the preoccupation with productivity stemmed not from economic motives but from professional pride. The department manager made clear (quote 3) that the urge to ‘produce’, was not imposed on the team by her. In fact, she preferred lower production rates, as overproduction was not being paid for.

#### 3.1.3 Avoiding conflict and pursuing conflicting priorities

A third characteristic of the team was the tendency to avoid conflict while pursuing conflicting priorities. The value placed on team cohesion, especially between surgeons and operating room nurses, was significant (quote 4). The core team routinely engaged in small talk to strengthen bonds, fostering numerous personal connections among surgeons and surgical nurses. Surgeons expressed a reliance on nurses for smooth and efficient processes (quote 5). However, the focus on maintaining affective relationships led to tension when team members faced conflicting priorities.

### 3.2 Preventing or responding to time pressure

#### 3.2.1 Creating Situational Awareness

Team members endeavoured to avoid conflicts regarding their priorities when possible. Vignette 3 illustrates how, on one occasion, time pressure was alleviated through the initiative of Nurse Jennifer, who addressed the conflict and fostered shared SA regarding workload planning. To mitigate time pressure, nurses proactively raised awareness of missing materials during the briefing.

Surgeon George had his own routine to preventing time pressure. He adhered to a long-standing personal habit of calling the nurses the day before to inform them of the materials and instruments he would require. Vignette 4 highlights how he established SA, control, and pacing for himself during procedures. The spinoff was that he created SA for the team as well, effectively preventing time pressure. When the researcher asked the core team why George's practice was not emulated by his colleagues or residents, they shrugged and remarked that this was characteristic of George—a maverick.

#### 3.2.2 Skipping new routines and adhering to established routines

We observed that the new briefing routine was frequently compromised by surgeons, despite their general trust in the judgement of the nurses and anaesthetists, who affirmed that the briefing saved time and enhanced SA and patient safety. In contrast, the established routine of weekly morbidity and mortality meetings was consistently conducted and usually attended by all residents and most consultants, even though participants did not regard them as particularly informative (quote 6). These meetings were mandatory for all departments involved in accredited training programmes for interns and residents, though their frequency varied by department. The established time-out procedure before incision was also always executed, regardless of its perceived contribution to safety (quote 7).

## 4 Discussion

This paper emerges from findings indicating that frontline workers often cite time pressure as a barrier to achieving quality and safety improvement goals at the team level. Therefore, the primary aim of this study was to explore the concept of time pressure and its interaction with the development of mindful routines.

### 4.1 Understanding time pressure

We illustrated how the team carefully fostered affective relationships by respecting and accepting that some colleagues are late adopters (vignette 2), carefully navigating sensitive topics (vignette 3) providing personal attention to all members of the operating room team (vignette 4), and engaging in small talk and private connections. Lingard et al. [22] describe this careful interrelating in the operating room as: ‘a complicated “dance’’ that maintains relationships and minimises tension while still achieving goals.’ Edmondson [23] suggests that strong affective relationships enhance the willingness to assist one another, contributing to the psychological safety necessary for sharing information across professional and hierarchical boundaries.

However, our findings suggest that this approach also inhibited team members from addressing conflicting priorities and signalling time-related issues. “It had to be in your character and your age” (vignette 3) to dare addressing the issue of ending in time. This distinguishes careful from heedful relating. Careful relating aims to establish affective relationships. Heedful relating [24] aims to connect distributed activities and information in which individuals subordinate their personal interests (such as avoiding conflict) to those of the system. The more heedfully the interrelating is done, the more capable of intelligent action the collective mind is [24]. Collective mind conceptualized as a pattern of actions driven by connected distributed knowledge.

Both Nurse Jennifer and Surgeon George fostered SA through their unique styles of heedful relating, effectively mitigating time pressure. Yet, these approaches were perceived as privileges associated with their positions, rather than as exemplary practices. Such a culture sustains time pressure.

We conclude that time pressure did not stem from a workload imposed by management; rather, it was co-created through the pursuit of differing priorities while maintaining affective relations. Therefore, addressing conflicting priorities and practising heedful relating sometimes alleviated time pressure, though these practices were not standard.

Further qualitative research is needed to explore how teams can develop skills to address conflicting priorities, engage in heedful relating, and develop collective mind.

### 4.2 Time pressure as a motivator for mindful routines

Our observations revealed that the team was preoccupied with time, speed and productivity [vignette 1, 2 and 4, quote 1, 2]. Several studies suggest that this is based in the surgical tradition of which anaesthesia is a branch [25,26]. It is a deep structure that ‘shapes organizational life because they manifest through practices that are routinised, and are continuously re-enacted over time’ [27]. Research on the uptake of a briefing or the WHO surgical checklist also identifies time pressure as a barrier to conducting briefings with all participants [28,29]. While surgeons were inclined to bypass briefings under time pressure, many were also willing to engage in the briefing because it was perceived by the nurses to save time (Table 3). The nurses connected this new routine to the existing deep structure by emphasising its potential for time savings later in the day.

**Table 3:** Differences causing conflicting priorities.

Differences in:	surgeons	Surgical nurses	Aesthetic team
Norms	Safety needs a skilled surgeon	Safety needs a briefing	Safety needs a briefing
Worries	Being perceived as slow and unproductive A cancelation conversation with the patient	Working overtime	Being perceived as slow and unproductive Irritated surgeons about delays at 8am Patient safety
Schedules	working day ends at 6pm Briefing starts at 8.00am	working day ends at 4pm Briefing starts at 8.00am	working day ends at 6pm First briefing is at 8.00am, second briefing at 8.05am
Experiences	Briefing costs time	Briefing saves time, because of early detection of missing materials. It manages time by agreeing on an evaluation moment.	Briefing saves time, because of early detection of missing materials. It manages time by agreeing on an evaluation moment.

We conclude that within surgical teams, (preventing) time pressure acted as a motivator for creating SA and adhering to mindful briefing routines.

At the organization level this might be different though. At this level maximum working hours, CRM training programmes, obligatory morbidity and mortality meetings, redundant staffing are secured. Other studies indicate that a preoccupation with productivity or profitability at the organisational level can undermine the conditions necessary for effective functioning in the operating room [[30], [31], [32]].

### 4.3 Habits for withstanding time pressure

Our results suggest that once a habit is established - such as the time out (quote 7) or the morbidity and mortality meeting (quote 6)- the original rationale for the habit may become irrelevant to its execution, and time pressure ceases to be a threat. Neal et al. [33] state that habits are not influenced by people's goals. Even moderately strong habits require substantial conscious effort to change [33]. Developing new habits require environmental cues that trigger the habitual behaviour, repetition, and socialising processes [20,[33], [34], [35], [36]].

The process of socialising into a profession takes time. As noted by Resident Jorin (vignette 2), the anaesthetic team had developed differing convictions regarding safety and excellence compared to surgeons (Table 3). In the Netherlands, these beliefs have been embedded in anaesthetic training for decades. An article discussing the evolution of the patient safety movement highlights the long-standing connection between anaesthesiology and patient safety [37]. Yet, as indicated by Bernhard's quote (vignette 1), not all anaesthetists have yet altered their unconscious convictions.

Creating cues to draw attention to patient safety risks requires less time than changing ingrained social behaviours. All elements of the new briefing routine pertained to patient safety risks. However, signalling the same risks for identical procedures daily can feel like "ticking boxes," leading to procedural decay [38]. Thus, we hypothesise that merely creating cues to signal risks is insufficient to support a consistent daily briefing. The immediate reward of saving time—a strong preoccupation receiving considerable conscious attention—contributed to the establishment of the new routine, alongside the fact that the routine was cued and repeated daily. The subsequent challenge is to execute all aspects of the briefing attentively and to prevent procedural decay, as noted by core team members (vignette 2) and supported by other studies [28,39].

It is practically relevant for designing routines, that the likelihood of consistent performance increases when a routine is positively associated with a preoccupation.

The observation that the established routine of morbidity and mortality meetings was not highly valued aligns with findings from other studies [40,41]. Nonetheless, this existing routine is performed automatically, even under time pressure, as it does not demand significant conscious attention (bandwidth) [20,21,42]. This presents an opportunity to optimise the routine. Promising initiatives regarding morbidity and mortality meetings include involving patients in discussions, conducting online meetings, and evaluating successful procedures through resilience concepts [41,[43], [44], [45]]. These interventions have altered participation dynamics and broadened perspectives on quality of care.

The practical implication is that existing morbidity and mortality meetings can be enhanced by incorporating interprofessional participation—potentially including patients—to address preoccupations and conflicting perspectives, thereby fostering the development of collective mind. This will encourage daily heedful behaviours and a shared valuation of routines.

Further longitudinal qualitative research is needed to understand how teams can optimise the mindfulness of existing routines or routines in the making.

## 5 Conclusion

In summary, time pressure was not a result of workload imposed on the team but rather emerged from a co-creative process involving conflicting priorities and maintaining affective relations. The drive to save time acted as a motivator for cultivating SA and establishing a new mindful routine. Established routines appeared resilient to time pressure. We recommend optimising mindful routines by refining existing morbidity and mortality meetings to include a broader range of stakeholders and to address time-related concerns.

## 6 Limitations

We studied a single team in-depth with ethnographic methods as part of a case study research design. Therefore, we could only draw conclusions about this specific case and formulate questions for further research. To enhance the transferability of the study we used extensive quotes and thick descriptions of real-life situations in the vignettes. According to the literature on case study research designs [16,46] this enables a vicarious experience in the readers, especially operating room professionals, enabling them to recognise the situations of time pressure and teamwork and translate them to their own specific context. As Simons [46] argues: “the overarching justification for how we learn from case study is particularization – a rich portrayal of insights and understandings interpreted in the particular context”. Furthermore, in formulating our practical implications about using existing routines, we drew not only on our findings but also on relevant evidence from other studies thereby contextualizing the local findings [17].

## CRediT authorship contribution statement

**Annet van Harten:** Writing – original draft, Project administration, Formal analysis, Data curation, Conceptualization. **Theo J.H. Niessen:** Writing – review & editing, Methodology, Formal analysis. **Jur J. Koksma:** Writing – review & editing, Methodology, Formal analysis. **Hein G. Gooszen:** Writing – review & editing, Formal analysis. **Tineke A. Abma:** Writing – review & editing, Supervision.

## Ethics and consent

Review and/or approval by an ethics committee was not needed in this study because it is not medical scientific research in which individuals are subjected to acts or rules of conduct. Therefore the Medical Research Involving Human Subjects Act (Wet op Medisch Onderzoek, WMO) did not apply to this project.

All participants were informed orally that consent to participate in the study and publish their data would be assumed on completion and submission of the study. Written consent from all participants was impractical when observing in the OR because it was often unpredictable who would participate and participants did not appreciate to read information and consent forms just before the start of an operation. Furthermore, some participants in the training shared that giving written consent was meaningless for them since they were unfamiliar with this type of qualitative research. The names of the participants in the narratives are fictitious to ensure anonymity.

The members of the core team and the interviewed stakeholders gave written consent for audio recording and quoting. The core team members checked the interpretation of the data on behalf of all participants in the last meeting of the research.

## Data and code availability

The data that has been used is confidential.

## Funding statement

This research did not receive any specific funding

## Declaration of competing interest

The authors declare that they have no known competing financial interests or personal relationships that could have appeared to influence the work reported in this paper.
