# Supplementary material for: Time pressure in surgical teams, a help or a hindrance to patient safety?
Source: Heliyon. 2025 Jan 15;11(2):e41967. doi: 10.1016/j.heliyon.2025.e41967 (PMC11786866; doi:10.1016/j.heliyon.2025.e41967)
Supplement: Multimedia component 1 [file mmc1.pdf]

Appendix S1: Additional information on methods

In this appendix we provide more detailed methodological information in addition to the methods paragraph in the article "Time pressure in surgical teams improving patient safety,
A naturalistic case study in a Dutch academic hospital". We do not repeat what is already described in the article.

Setting

The anesthetic disciplines participate in the team, but are not dedicated to one surgical specialty. The nurses work mainly in this surgical specialty, but all have a second surgical specialty in which they work part of the week as well. Most other surgical specialties had already started with Crew Management Resources and with briefings at the start of the day.

Figure 1. Shows the items of the briefing

Figure 1 briefing and debriefing checklist for the OR

BRIEFING OR	DEBRIEFING OR
**Personnel**<br>- Are we complete? Does everyone know each other?<br>- Are functions and expertise clear?<br>- Are there specific learning goals? (eg: performing double check, less door movements)	**Personnel**<br>- What went well in teamwork, what can be done better?<br>- How did we do on our learning goals?
**Patients**<br>- Are there surgical particulars?<br>- Estimated surgical time, risks, problems?<br>- Materials, instruments, apparatus<br>- Specific applications made? (e.g. rontgen)<br>- Anaesthesiologic particulars?	**Patients**<br>- Were procedures performed well, learning points, incidents, improvement suggestions?
**Planning**<br>- If deviant staffing: who is doing what?<br>- Who coordinates planning of the day?<br>- When do we do the debriefing?	**Planning**<br>- Logistic improvement goals?<br>- Who notes improvements suggestions and where?

Researcher reflexivity

I have a background in organization psychology and change management and worked for many years as a management consultant and did so several years in the academic hospital where the research took place. My theoretical orientation has always been a constructivist one. I had done many projects in the operating rooms and as such was familiar with culture, procedures and safety rules in several disciplines.

The medical heads of the surgeons, the nurses and the anesthetists - three different departments - gave me the assignment for the implementation of Crew Resource Management (CRM) and also consented in participating in the research, just like the core team and the participants. They agreed that I and the chair of the core team would primarily report progress to the management of the surgeons (medical head and manager). When desirable, the other heads could be contacted.

In my role as CRM trainer I delivered the training offering a mix of theory, discussion and exercises.

The training was given in groups of approximately 15 persons, nurses, anaesthetists and surgeons together. The training was given together with a co-trainer, to enable me to listen and observe more attentively.

In my role as facilitator, I tried to leave the initiative in the core team. I intervened by introducing theoretical concepts and by mirroring my observations and reflections. It was up to them to decide on next steps.

In my role as researcher, I could interview and observe whenever I felt that was useful. Members of the core team were mainly curious or sceptic about the ethnographic methodology of the research. In their view (medical) science requires countable data and statistical analysis. But they were willing to support the research and, working in an academic hospital, they were used to being observed and to participate in a study. The core team showed little awareness of the research side of their project during the meetings.
The research question was formulated together with the core team and was formulated initially as "how can we implement the intended behavioral improvements leading to situational awareness (SA)?" The behavioral improvements were specified as performing the briefing well and speaking up. However, during the research most energy went to implementing the briefing. The theme of time pressure emerged during the process. It was a dominant topic in almost all meetings of the core team.
The combination of facilitating the core team and observing, was most challenging. Part of the observations came into awareness after the meeting when making the fieldnotes or transcriptions from the audio recordings or in the bi-weekly reflections on the fieldnotes.

Trustworthiness of the research

To enhance the trustworthiness of the research [1], the following procedures were used.

Table 1 trustworthiness of the research

Quality criteria	realization
Credibility	*Prolonged engagement:* The prime researcher (XXX) spent 18 months in the research setting and was familiar with the larger context of the operating room as a consequence of other projects being carried out. Co-researcher (XX) worked as a surgeon in other hospitals for many years and as the head of the operating room department of this hospital at the time. This prolonged engagement enabled the researchers to collect persistent and reliable observations. Because of their prolonged engagement in many surgical teams, they were sensitive to standard practice and deviations from that standard.
	Researcher reflexivity: During the data collection, the participant observer reflected every two weeks with dr XXX, a professor on medical education to stay as open-minded as possible. They reflected on the data, her thoughts, assumptions, feelings, role of participant observer and the way she influenced the course of events and the reactions of the participants.
	Member checking (respondent validation): Members of the core OR team were asked to give their comments on the story and the interpretation presented
	Transparency: by adding this appendix we provide detailed information on the process of the research.

	Method triangulation: data were obtained from: open interviews, informal conversations, observations, participatory meetings such as core team meetings and trainings.
	Theory triangulation: using different theorical angles to interpret the phenomena
	Researcher triangulation: five authors and one additional researcher from different backgrounds were involved in analyzing the data, to ensure different perspectives and interpretations. The backgrounds of the authors were: change management and psychology, medical humanities, educational sciences, surgery, nursing studies
Transferability	rendering thick descriptions [2] in vignettes evoking 'vicarious experiences' [3] combined with quotes. The vignettes were selected because of their learning potential, they illustrate a dynamic or mechanism [4].
Confirmability and dependability	All authors read and analyzed raw data such as transcripts and fieldnotes individually. They discussed issues until consensus was reached on the selection of the most important data fragments, interpretations and themes. We described the research design and data collection in detail.

Analysis

As described in the paper, we performed a thematic analysis at various points in the project by reading and rereading the data and discussing them in the research team, thinking with theory [5]. In table 2 we provide more details on the procedure and content of the analysis.

Table 2 process of the analysis

phase	Themes and topics
First diverging phase (halfway data collection)	We all recognized time and time pressure as a dominant theme.<br>The first author deepened our understanding of time pressure by reading sociological and philosophical literature on time and temporality, the societal context and tolerance for safety risks influencing the experience of time pressure.<br>Other themes: boundary crossing, power relations and gender issues in building time pressure in the team.
Second diverging phase	We discussed the learning and reflective practices in handling time pressure in the operating team as well as in the facilitator.<br>we chose an emic descriptive perspective by reflecting on the concrete experiences of time pressure for each discipline in the operating team and an etic interventionist perspective oriented at change and development of behaviors leading to situational awareness in a complex organizational context with many interdependencies.
Converging phase	We chose to stay close to the strong concrete ethnographic descriptions that can evoke a vicarious experience in the reader who will often struggle with time and projects as well.<br>We interpreted the descriptions from an interactionist perspective. The vignettes and quotes show the interactively constructed nature of time pressure and the motives or drivers that guide the behaviors that build and resolve time pressure.

	To hypothesize about the relation to improving mindful organizing we chose for the concepts of habit and thus for the perspective on humans as habitual entities in a relational system.

1. Lincoln YS, Guba EG: *Naturalistic inquiry*. Beverly Hills, Calif.: Sage Publications; 1985.
2. Shenton AK: **Strategies for ensuring trustworthiness in qualitative research projects**. *Education for information* 2004, **22**:63-75.
3. Abma TA, Stake RE: **Science of the Particular: An Advocacy of Naturalistic Case Study in Health Research**. *Qual Health Res* 2014, **24**:1150-1161.
4. Anderson PJJ: **Understanding Mechanisms in Organizational Research: Reflections From a Collective Journey**. *Journal of Management Inquiry* 2006, **15**:102-113.
5. Jackson AY, Mazzei LA: **Plugging One Text Into Another: Thinking With Theory in Qualitative Research**. *Qualitative Inquiry* 2013, **19**:261-271.
